# Ways to Improve the Efficiency of Devices for Freezing of Small Products

**DOI:** 10.3390/ma15072412

**Published:** 2022-03-25

**Authors:** Oleg Bazaluk, Nikolai Struchaiev, Serhii Halko, Oleksandr Miroshnyk, Larysa Bondarenko, Oleksandr Karaiev, Vitalii Nitsenko

**Affiliations:** 1 Belt and Road Initiative Institute for Chinese-European Studies, Guangdong University of Petrochemical Technology, Maoming 525000, China; bazaluk@ukr.net; 2 Department of Information Technologies of Design, Dmytro Motornyi Tavria State Agrotechnological University, 18 B.Khmelnytsky Ave, 72310 Melitopol, Ukraine; mykola.struchaiev@tsatu.edu.ua (N.S.); serhii.halko@tsatu.edu.ua (S.H.); larysa.bondarneko@tsatu.edu.ua (L.B.); oleksandr.karaiev@tsatu.edu.ua (O.K.); 3 Department of Electricity and Energy Management, State Biotechnological University, Str. Rizdviana, 19, 62341 Kharkiv, Ukraine; omiroshnyk@ukr.net; 4 SCIRE Foundation, 00867 Warsaw, Poland

**Keywords:** mechanized device, small-piece, fluidized bed, quick freezing, fruit and vegetable, energy savings, experimental studies, thermal conductivity, equivalent diameter, time of pre-freezing, heat capacity, energy consumption, cascade freezer, result, graph, analysis

## Abstract

It has been established that one of the main problems in the technology of the production of loose food products is the sticking of vegetables or fruits into one block. It has been proven that one of the steps to solve this problem is the use of berries, fruits, or vegetables during freezing in the form of a fluidized bed in air. However, a significant part of the energy is spent precisely when creating a fluidized bed with the help of fans. By improving the separation efficiency of small products in the freezing process, it would be possible to significantly reduce the energy costs of freezing worldwide. The purpose of this work was to determine ways to increase the efficiency of devices for freezing small products. The goal was achieved through the use of a modified method for studying energy costs, taking into account energy costs for fluidization and mechanical shaking. For comparison, two options for the efficient separation of small products during freezing were considered. Namely the separation of small products in the process of freezing with the help of fluidization, and with the help of mechanical shaking. Comparison of these variants showed that it was advisable to separate small products during freezing by mechanical shaking. It was established that their energy parameters, as well as fractional properties, are significantly different. The product temperature was determined for the case of a constant temperature of the cooling air and equipment elements. The results obtained confirmed the possibility of achieving significant energy savings of 1.5–3.5 times by using the mechanized device we proposed for freezing fruits and vegetables. The main result of this paper is the proposed method, or algorithm, for calculating energy costs for fluidization and mechanical shaking, which could be used in the design of devices for the freezing of small products; as well as the obtained data confirming the correspondence of the theoretical calculations to reality. The novelty of the research consists in presenting a model or algorithm for calculating the energy costs for fluidization and mechanical shaking. The importance of the results of the work lies in the possibility of using this technique to assess the energy effectiveness of devices for the freezing of small products.

## 1. Introduction

In terms of energy savings and increased environmental security, being grown seasonally in the open ground, fruit and vegetable products become the main source of nutrients and vitamins [1,2,3,4]. Long-term storage of fruits and vegetables is currently a very pressing problem. Freezing is one of the most effective ways of prolonging the use of fruits and vegetables beyond the harvesting period; the technological regimes and conditions of which are constantly being improved [5,6]. Freezing is one of the most widely used methods of food preservation because of two main reasons: The first is that many microorganisms cannot grow at the low temperatures used in freezing [7]. In addition, when a food is frozen, part of the water is transformed into ice, thus decreasing the food’s water activity [8]. This decrease influences the growth of many microorganisms, since they cannot develop in low water activity conditions [9,10,11].

The effect of the freezing process on the state of plant cells can vary, depending on the size of the ice crystals formed at different freezing rates. One method of progressive is to improve the freezing technology. An effective method is to freeze small pieces of products in a dense weighted layer using rapid freezing. In all the freezing processes in this work, strawberries were frozen in a perforated stainless steel container [12]. This method allows speeding up the process, in combination with an increased simplicity in the sanitary-hygienic treatment of the equipment [13,14,15].

Considerable attention has been paid to the thermophysical aspects of the freezing of fruit and vegetables. The coefficients of thermal conductivity of different fruits during freezing were determined. The empirical dependences for determining the amount of heat during freezing and thawing and other thermo-physical calculations were obtained [16,17,18]. Of considerable interest are the studies on the prevention of freezing of the layer of fruits and berries, by pre-freezing them in a layer or using fluidization devices [19,20].

The weakness of the existing processes and devices is the significant energy consumption for fluidization, and the removal of moisture from the product due to high speeds (2.7–3.9 m/s) of the air that blows the berries or fruits. In addition, if you have seen how the fluidization apparatus works, then of course you have noticed that the flow of strawberries just flops up and down all the time. At the same time, the berries are beating against each other and against the conveyor [20]. When using a cascade quick freezer, the thickness of the freezing layer can be adjusted to one berry, cube, or slices.

Analytical and experimental studies are presented in [5,21,22,23,24,25]. However, it should be noted that the comparative study of energy consumption when using fluidization devices and mechanical or semi-fluidization quick-freezers has received little attention [10,26,27,28,29,30].

This is why it is absolutely necessary to reduce the energy consumption for creating a fluidized bed, as well as to reduce the time in the preliminary stage, needed to create an ice crust.

In fluidization freezing, the freezing of the fruit is due to the flow of cold air, while in mechanical or pseudo-fluidizing freezing devices, the fruits are constantly moved by fluctuations in the working surface of the conveyor.

The purpose of this work was to determine ways to increase the efficiency of devices for freezing small products. The tasks of the article include: to compare the cascade freezer, we offer an option for improving refrigeration processing equipment for freezing small-scale products in a dense suspended layer using quick-freezing devices with fluidizing devices. The task is to simplify the design, reduce energy consumption, improve the stability of the layer of suspended frozen product, and increase the rate of heat transfer in the fluidized bed and the performance of the freezing process as a whole.

The following positive effects are expected from the proposed method:

Reducing energy consumption for creating a pseudo-fluidizing layer, reducing the time for the preliminary stage needed for creating an ice crust, and a more delicate pouring of the product into a mesh drum until a protective ice crust has formed.

## 2. The Basic Part of the Study

### 2.1. Model Representation of the Problem and Its Solution

In this section, we present a model or algorithm for calculating energy costs for fluidization and mechanical shaking.

The calculation results will be compared. The comparison will show which method of freezing is energetically more beneficial.

We determine the energy costs of fluidization and mechanical shaking.

To perform calculations, first of all, we determine the energy consumption for fluidization using the Formula (1):

(1) $$E_{fl}=V\cdot \Delta P\cdot F,$$

where

$E_{fl}$—energy consumption for fluidization, W;

V—operating air velocity, m/s;

ΔP—pressure drop when moving air through the fruit layer, Pa;

F—the cross-sectional area, m^2^.

The following quantities are unknown in Equation (1): pressure drop when air moves through the fruit layer, air velocity, and cross-sectional area. Let us define them one after another.

Therefore, the pressure drop in the layer is determined by Formula (2):

(2) $$\Delta P=\left(\rho_{f}-\rho_{air}\right)\cdot g\cdot \left(1-\varepsilon \right)\cdot H,$$

where

$\rho_{f}—$the density of the layer of fruits, kg/m^3^;

$\rho_{air}—$air density, kg/m^3^;

ε—porosity of the fluidized bed;

H—layer height, m.

Let us clarify the pressure drop in the layer. The pressure drop in the fluidized bed is more specifically determined by the Ergan equation, taking into account the viscosity of the liquid or gas phase and the equivalent diameter of the fruit. The pressure drop in the layer according to the Ergan equation [31,32,33] is determined by the Formula (3):

(3) $$\Delta P=150\cdot \frac{{\left(1-\varepsilon \right)}^{2}}{\varepsilon^{3}}\cdot \frac{\mu \cdot V}{d_{e}^{2}}\cdot H+1.75\frac{1-\varepsilon_{0}}{\varepsilon_{0}^{3}}\cdot \frac{\rho_{f}\cdot V^{2}}{d_{e}}\cdot H,$$

where

μ—the viscosity of the liquid or gaseous phase, Pa·s;

$d_{e}$—the equivalent diameter of the fruit, m;

$\rho_{f}—$the density of the layer of fruits, kg/m^3^.

Now, let us start by determining the air speed. To determine the air speed, we first find the Reynolds criterion and the Archimedes criterion using the criterion dependences of O.M. Todes [34,35].

The Reynolds criterion is determined by Formula (4):

(4) $$Re=\frac{Ar}{1400+5.22\sqrt{Ar}},$$

where

*Re*—Reynolds’ criterion;

*Ar*—Archimedes’ criterion.

However, to calculate the Reynolds criterion, you must first calculate the Archimedes criterion. Archimedes’ criterion is determined by Formula (5):

(5) $$Ar=g\frac{d_{e}^{3}}{\nu^{2}}\cdot \frac{\rho_{f}-\rho_{air}}{\rho_{air}},$$

where

*g*—acceleration of gravity, m/s^2^;

*d_e_*—equivalent fruit diameter, m;

ν—the kinematic viscosity of the air, m^2^/s;

$\rho_{f}—$the fruitdensity, kg/m^3^.

$\rho_{air}—$the air density, kg/m^3^.

Taking into account Formulas (4) and (5), the air speed at the beginning of fluidization is determined from expression (6):

(6) $$V_{0}=\frac{Re\cdot \mu }{d_{e}\cdot \rho_{air}},$$

where

$V_{0}—$the air velocity at the beginning of the fluidization, m/s,

μ—dynamic viscosity of air, Pa·s;

Operating air speed is determined by Formula (7):

(7) $$V=2\cdot V_{0},$$

where

V—operating air velocity, m/s.

To carry out calculations to determine the energy consumption for fluidization according to Formula (1), it is necessary to carefully perform all calculations according to Formulas (2)–(6). Then, substitute the found values of air velocity and pressure drop when air moves through the fruit layer into Formula (1). One also needs to find out the cross-sectional area of your equipment, according to the technical data sheet and also insert it into Formula (1).

At the second stage, determine the energy costs for the mechanical tossing of berries or fruits.

The energy costs for mechanical tossing by a mesh conveyor, is determined by Formula (8):

(8) $$E_{m.con}=\frac{4\cdot \left(M_{m.con.}+M_{pr}\right)\cdot \pi^{2}\cdot n^{3}\cdot A^{2}}{10.2\cdot g},$$

where

$E_{m.con}$*_._*—the energy costs for mechanical tossing by a mesh conveyor, W;

$M_{m.con.}—$the mass of the mesh conveyor, kg;

$M_{pr}—$the mass of frozen product, kg;

n—oscillation frequency, 1/s;

A—oscillation amplitude, m.

We determine the mass of the mesh conveyor by the Formula (9):

(9) $$M_{m.con}=10\cdot m_{g}\cdot z_{n.g.},$$

where

$m_{g}$—the mass of one grid, kg;

$z_{n.g.}—$the number of grids in the mesh conveyor, pc.

Furthermore, the mass of frozen product is determined by Formula (10):

(10) $$M_{f}=\rho_{pr}\cdot v_{pr},$$

where

$M_{pr}—$the mass of frozen product, kg;

$\rho_{pr}—$the density of the layer of product, kg/m^3^;

$v_{pr}—$volume of the product, m^3^.

Then, the oscillation frequency of the mesh conveyor *n*, 1/s, is determined by Formula (11):

(11) $$n=\frac{1}{\;20}\cdot \sqrt{\frac{10\cdot j_{0}}{A}},$$

where

n—the oscillation frequency of the mesh conveyor, 1/s;

$j_{0}—$optimum acceleration of the mesh conveyor, m/s^2^;

A—oscillation amplitude, m.

Finally, the amplitude of vibrations of the mesh conveyor A, m, is determined by Formula (12):

(12) A=e·k,

where

A—the oscillation amplitude of the mesh conveyor, m;

e—eccentricity, m, (e = (5... 10)·10^−3^ m);

k—coefficient taking into account the oscillations of the frame of the machine, depending on the optimal acceleration of the mesh conveyor.

To carry out calculations to determine the energy consumption for mechanical tossing according to Formula (8), it is necessary to carefully perform all calculations according to Formulas (9)–(12). Then we substitute the found values into Formula (8). You also need to find out the characteristics of your equipment according to the technical data sheet and also insert them into the Formula (8).

Having carefully performed all the calculations according to Formulas (1)–(12), it is possible to build graphs. Compare the results obtained and draw conclusions. Namely, we will compare the results of calculating the energy consumption for fluidization and mechanical throwing.

For example, in the results and discussion section, a comparison is made that will show which method of freezing is energetically more beneficial.

Another of the main problems of the technology of freezing juicy food products, such as sliced vegetables, berries, or fruits, is their adhesion during freezing. One of the steps to solve this problem is to pre-freeze them to create a protective layer of ice on the surface of the product pieces during freezing.

Pre-freezing is the process of lowering the temperature of products below cryoscopic, in which partial crystallization of moisture in the surface layer occurs. That is, when exposed to low temperature, ice formation occurs on the surface of the products; in our case, this is a preliminary operation, preventing the juicy fruits, berries, and chopped vegetables from sticking together. The product is placed in a fluidization freezer or a mechanized small-piece freezing device that we designed, where the peripheral layer of the product is frozen to a limited depth; and in the next stage, freezing is performed to the temperature specified by the technology.

**Figure 1:**
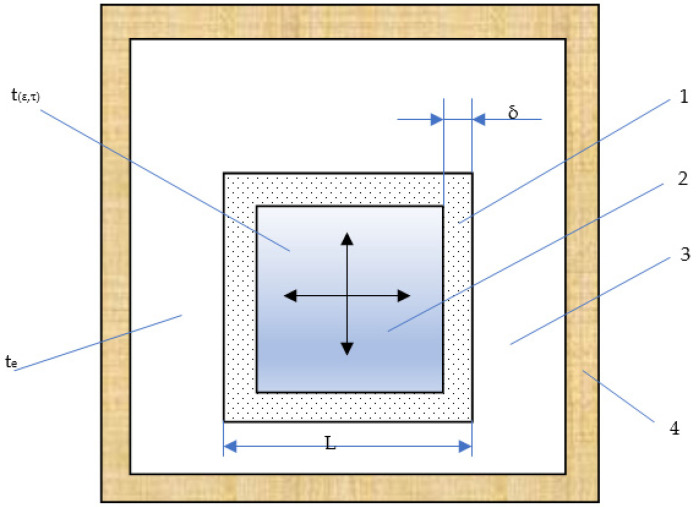
Product cube freezing model: 1—frozen layer, 2—layer of the product that will be frozen, and which has an initial temperature, 3—cooling air, 4—thermal insulation of a mechanized device for small-piece freezing.

The model for freezing the product cube can be represented in the form of a schema (Figure 1).

When pre-freezing a cube of small-piece product, the amount of heat that needs to be removed through its faces is denoted by: $dQ_{x}$, $dQ_{y}$, $dQ_{z}$. Then, taking into account the time of freezing, we obtain the equation:

(13) $$dQ_{x}=q_{x}\cdot dy\cdot dz\cdot d\tau ,\;dQ_{y}=q_{x}\cdot dx\cdot dz\cdot d\tau ,\;dQ_{z}=q_{x}\cdot dy\cdot dx\cdot d\tau ,$$

where

d—the projection of the heat flux density along the corresponding axis, J/(m^2^·s);

τ−the time of freezing, s.

The amount of heat allocated from the volume of the cube is found using the formula:

(14) $$dQ_{pr}=-\left(\frac{\partial q_{x}}{\partial x}+\frac{\partial q_{y}}{\partial y\;}+\frac{\partial q_{z}}{\partial z}\right)\cdot dx\cdot dy\cdot dz\cdot d\tau ,$$

where

$Q_{pr}-$the initial amount of heat in the product, J.

The amount of heat due to the specific heat contained in the cut element of vegetables is found using Formula (15):

(15) $$dQ_{ch}=c_{pr}\cdot p_{pr}\frac{dt}{d\tau }\cdot dv_{pr}\cdot d\tau ,$$

where

$Q_{ch}-$the amount of heat due to the specific heat contained in the cut element of the product, J;

$c_{pr}-$the heat capacity of the product, J/(kg·K);

t —temperature in the product, K;

$v_{pr}-$volume of the product, m^3^.

The boundary conditions for the case of freezing a cube of a juicy product in air can be represented as (16):

(16) $$\lambda {\left[\frac{\partial t\left(\varepsilon ,\tau \right)}{dz}\right]}_{i.c.}+\alpha \cdot \left[t_{i.c.}\cdot \tau_{i.c.}-t_{e}\cdot \tau_{e}\right]+h\cdot U\left(\tau \right)=0,$$

where

λ—the thermal conductivity of the product, W/(m·K);

α— the heat transfer coefficient from the surface of the product, W/(m^2^·K);

$t_{i.c.}—\;$initial temperature in the product, K;

$t_{e}—\;$the initial ambient temperature, K;

$U\left(\tau \right)—$the drying rate, kg/(m^3^·K);

h— the specific heat of vaporization, J/kg.

To determine the temperature distribution over the thickness of the product cube and the thickness of the frozen layer $\left(\delta \right)$, with specific heat removal $\left(q\right)\;$and freezing time $\left(\tau \right)$, we apply the Laplace transform and obtain the solution for the original (17):

(17) $$t_{i.c.}-t\left(\varepsilon ,\tau \right)=\left[t_{i.c.}+\frac{\rho \cdot U^{//}}{\alpha }-t_{\left(e\right)0}\right]+\frac{h\cdot \left(U^{/}-U^{//}\right)}{\alpha }\times \left(\frac{m}{\alpha }\cdot R^{2}\cdot \frac{\alpha \cdot R}{\lambda }\cdot \frac{\varepsilon }{R}\right)\cdot \exp\left(-m\cdot \tau \right)-\;-\overset{\infty }{\underset{n=1}{\sum }}A_{r,H}\left[t_{i.c.}+\frac{h\cdot U^{//}}{\alpha }-t_{\left(e\right)0}-\frac{h\cdot \left(U^{/}-U^{//}\right)}{\alpha }\times \frac{\mu_{i.c.}^{2}}{m\cdot R^{2}-\mu_{i.c.}^{2}}\right]\times \Phi_{r,H}\cdot \left(\mu_{i.c.}\cdot \frac{\varepsilon }{R}\right)\cdot exp\left(-\mu_{i.c.}^{2}\cdot \frac{\alpha \cdot \tau }{R^{2}}\right)-\;-\overset{\tau }{\underset{0}{\int }}t_{e}^{/}\left(\tau -\theta \right)\times \left[1-\overset{\infty }{\underset{n=1}{\sum }}A_{r,H}\cdot \Phi_{r,H}\left(\mu_{i.c.}\cdot \frac{\varepsilon }{R}\right)\times \exp\left(-\mu_{i.c.}^{2}\cdot \frac{\alpha }{R^{2}}\cdot \theta \right)\right]\cdot d\theta ,$$

where

$t_{\left(e\right)0}\;$is the initial temperature of the cooling air, K.

For the case of a constant temperature of the cooling air and the absence of evaporation, the temperature of the product cubes can be found by Formula (18):

(18) $$\frac{t_{\left(\varepsilon ,\tau \right)}-t_{e}}{t_{i.c.}-t_{e}}=\left[\overset{\infty }{\underset{n=1}{\sum }}A_{r,H}\cdot \Phi_{r,H}\left(\mu_{i.c.}\cdot \frac{\varepsilon }{R}\right)\cdot \exp\left(-\mu_{i.c.}^{2}\cdot F_{0}\right)\right],$$

or using the Bio criterion, which takes into account the relationship between the transfer of heat from the surface of the product to the heat sink medium and vice versa, we can use the Formula (19):

(19) $$t=\frac{t_{ct}\cdot \left(B_{i}+2\right)+t_{e}\cdot B_{i}}{2\cdot \left(B_{i}+1\right)},$$

where

$B_{i}—$the Bio criterion:

(20) $$B_{i}=\frac{\alpha \cdot \delta }{\lambda_{pr}}$$

where

α—the heat transfer coefficient during freezing, W/(m^2^·K);

δ—half the thickness of the product, m;

$\lambda_{pr}—$thermal conductivity coefficient of the frozen product, W/(m·K);

$t_{ct}—$temperature in the center of the product, K;

$t_{e}—$the ambient temperature, K.

To determine the time *τ* of pre-freezing a layer of a certain thickness, we can use the modified Planck formula [20] (21):

(21) $$\tau \left(\Delta \right)=\frac{\Phi \cdot q\cdot \rho \cdot R^{2}\cdot w}{\lambda \cdot \left(t_{cr}-t_{c}\right)}\times \left[\left(\frac{1}{Bi}+\frac{\Phi }{2\Phi -1}\right)\cdot \left(1-{\left(1-\frac{\Delta }{R}\right)}^{\frac{1}{\Phi }}\right)-\frac{1-{\left(1-\frac{\Delta }{R}\right)}^{2}}{2\cdot \left(2\Phi -1\right)}\right],$$

where

Δ—the thickness of the frozen layer of the product, m;

Φ—the coefficient of the shape of the product;

q—the specific heat of crystallization of water, J/kg;

ρ—the density of the product; kg/m^3^;

R—the characteristic size of the product, m;

λ—thermal conductivity of the frozen part of the product, W/(m·K);

$t_{cr}—$the ambient temperature of the refrigerant product, K;

w—the moisture content of the product, kg/kg.

To determine the thickness of the frozen layer during the preparation of chopped cubes of vegetables, it is necessary to fulfill the condition that the amount of heat in the cube body is less than the amount of heat required to melt the freezing layer.

The condition for creating a pre-freezing layer of sufficient thickness to prevent their adhesion is written in the form of the heat balance, Equation (22):

(22) $$Q_{i.c.}-Q_{prefr}\leq Q_{mlt},$$

where

$Q_{i.c.}—$initial amount of heat in the product, J;

$Q_{prefr}—$amount of heat in pre-freezing layer of product, J;

$Q_{mlt}—$amount of heat to melt the frostbitten layer of product, J.

Furthermore, the amount of heat of the previously frozen layer of the product is determined by Formula (23):

(23) $$Q_{prefr}=c_{fr}\cdot \rho_{fr}\cdot V_{fr}\left(t_{i.e.}-t_{cr}\right),$$

where

$c_{fr}—\;$specific heat of the frozen product, kJ/(kg·K);

$\rho_{fr}—\;$density of the frozen product, kg/m^3^;

$V_{fr}—\;$volume of frozen product, m^3^;

$t_{i.e.}—$initial temperature in the product, K;

$t_{cr}—$cryoscopic temperature of the product, K.

The amount of heat for melting the frozen layer of the product is determined by Formula (24):

(24) $$Q_{mlt}=\lambda_{pr}\cdot \rho_{fr}\cdot V_{fr},$$

where

$\lambda_{pr}—$specific heat of fusion of the frozen product, kJ/kg.

In Formula (24), the volume of the frozen part of the fruit is unknown.

The volume of the frozen part of the product can be determined using the scheme presented in Figure 1, according to the Formula (25):

(25) $$V_{fr}=2\cdot L_{fr}^{2}\cdot \delta_{fr}+2\cdot L_{fr}\cdot \left(L_{fr}-\delta_{fr}\right)\cdot \delta +2\cdot {\left(L_{fr}-2\cdot \delta_{fr}\right)}^{2}\cdot \delta_{fr},$$

where

$L_{fr}—$linear cube size of the frozen portion of the product, m;

$\delta_{fr}—$thickness of the pre-frozen layer of the product, m.

The volume of the non-frozen part of the product can be determined using the scheme shown in Figure 1, according to the Formula (26):

(26) $$V_{n-fr}={\left(L-2\cdot \delta \right)}^{3}$$

Solving Equations (15), (18) and (22)–(26) together, with respect to the thickness of the layer that we freeze, we find the layer thickness that ensures that the cube surface of the frozen product does not thaw.

This value will ensure that the cubes are not frozen into a single block:

(27) $$\delta_{fr}=f\left(c_{i.e.},\rho_{i.e.},t_{i.e.},t_{cr},c_{fr},\rho_{fr},L,\lambda ,t_{\left(\varepsilon ,\tau \right)}\right)$$

For example the thermophysical characteristics of squash required for calculations are given in Table 1.

### 2.2. Materials and Methods

The research method is based on an improved method for studying the process of freezing juicy food products, such as sliced vegetables, berries, or fruits [15,16,17,28,29,30,31,32,33,34,35,36,37]. Two-stage freezing was used with the formation of a frozen layer (ice crust), which prevented the possible sticking of vegetable cubes when the internal moisture passed to the crystalline structure. For the formation of an ice crust in the first stage, a vessel with liquid nitrogen Kharkov-31 and a PPK-5 potentiometer were used. The juicy food products, such as sliced vegetables, berries, or fruits, in the second stage were finally frozen in air in a stationary refrigerating chamber, the temperature of which was maintained using two FAL-56 refrigerators. The temperature at the center of the juicy foods, such as sliced vegetables, berries, or fruits, was measured with a calibrated chromel-alumel thermocouple, electromotive force, which is 8.3 mV/100 °C. Before freezing, the berries were initially stored in a pre-cooling chamber at a temperature of 5–2 °C [28]. In a freezer with a mesh conveyor, forced air circulation was provided at a speed of 1.0–2.5 m/s at a relative humidity (φ) of 88–93%. The temperature at which the adhesion of pieces of squash in one block ceases was determined by the termination of the adhesion of a piece of product to the strain gauge beam. Freezing was considered complete when the berries were frozen to the full depth and reached a temperature of minus 20 ± 2 °C in the center of the berries. At this temperature, protein denaturation is significantly reduced, which creates optimal conditions for long-term storage [28].

## 3. Results and Discussion

**Figure 2:**
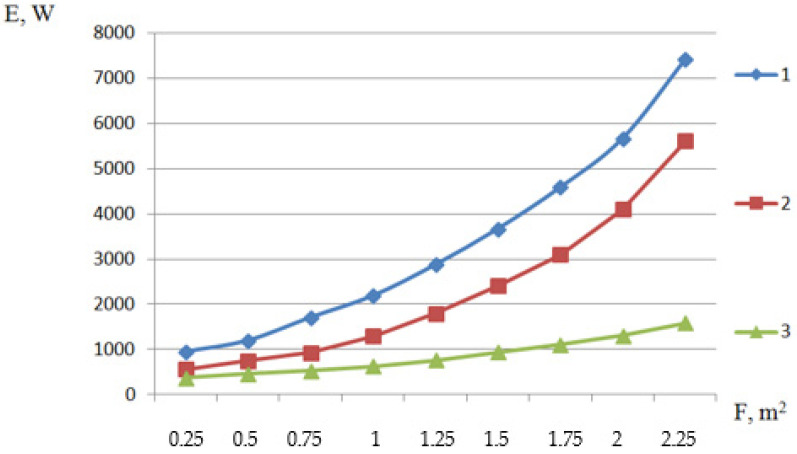
Dependence of energy E, (W) consumption on the cross-sectional area F, (m^2^) to create a fluidized bed and mechanical shaking when freezing raspberries; 1—fluidized bed at *V* = 3.9 (m/s), 2—fluidized bed at *V* = 2.7 (m/s), 3—mechanical shaking.

We will calculate the energy costs for creating a fluidized bed and for mechanical tossing of frozen raspberries. We accept the air velocity *V* = 2.7–3.9 m/s; the pressure drop when moving air through the layer of fruits ΔP=919 Pa; cross-sectional area $F=0.5\;m^{2}$; density of the layer of fruits $\rho_{f}=580$ kg/m^3^; the fluidity of the fluidized bed ε=0.4… 0.55; air density $\rho_{air}=1.42\;\mathrm{kg}/m^{3}$; layer height H=0.1… 0.27 m. The graph in Figure 2 shows that the energy costs for creating a fluidized bed compared to the mechanical shaking of frozen raspberries are 1.5–3.5 times greater.

**Figure 3:**
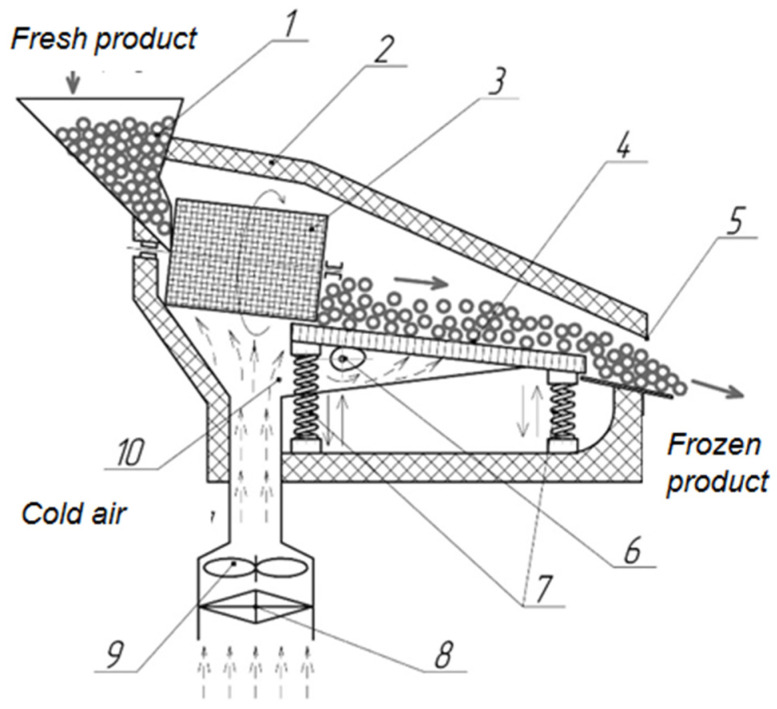
Functional scheme of the cascade freezer; 1—download block, 2—thermally insulated chamber, 3—a rotating hollow inclined drum made of mesh, 4—mesh conveyor, 5—unloading window, 6—eccentric mechanism, 7—springs, 8—evaporator of the refrigeration unit, 9—fan, 10—distribution channels of low temperature air.

Based on the results of the study, we received a patent for the cascade freezer that we proposed [27]. A functional diagram of the cascade freezer is shown in Figure 3.

The cascade freezer contains a download block, a thermally insulated chamber for freezing products with a mesh conveyor connected to the evaporator of the refrigeration unit through low-temperature air distribution channels, a fan, a mesh hollow rotating drum is inclined in front of the mesh conveyor, and the mesh conveyor is made in the form of vibratory conveyors with an eccentric mechanism and springs, while, at the outlet of the chamber is unloading window. The cascade freezer uses the evaporator of the refrigeration unit or stationary refrigerating chamber.

The application of the cascade freezer of the proposed design is as follows: In the freezing shop, the heat-insulated chamber (2) is installed for freezing the products, at the entrance to the chamber a download block (1) is installed; in the chamber (2), there is a rotating hollow inclined drum made of mesh (3), a mesh conveyor (4) with an eccentric mechanism (6), springs (7), and an unloading window (5). The chamber (2) is connected to the evaporator (8) of the refrigeration unit through channels (10) for distribution of low-temperature air and a fan (9) for supplying low-temperature air to the chamber (2). The product to be frozen is continuously fed to the download block (1) into the mesh hollow rotating drum (3), simultaneously turning on fan (9). The rotational motion and free falling of the product begins, with vigorous heat exchange with low temperature air, which causes the outer layer of the product to freeze. Next, the product is poured onto a mesh conveyor (4), where, under the action of the eccentric mechanism (6) and the springs (7), its vertical and horizontal movement begins and a fluidized bed is formed. The fan (9) through the evaporator (8) of the refrigerating unit and the channels (10) for air distribution supplies the cooled low-temperature air to the chamber (2). The air stream passes through the mesh conveyor (4) and, finally, freezes the product in the fluidized bed. From the mesh conveyor (4), the product is poured through an unloading window (5), from which it is fed into the packaging machine (not shown).

The joint solution of Equations (22)–(27), taking into account a more detailed distribution of thermophysical parameters versus temperature, is presented in Table 2.

**Figure 4:**
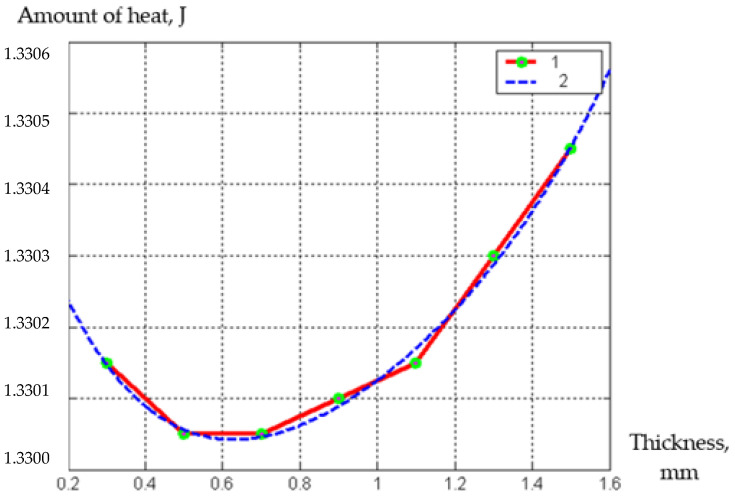
Dependence of the thickness (mm) of the frozen layer on the amount of heat (J) removed during preliminary freezing, 1—experimental curve, 2—approximating curve.

Solving Equations (13)–(27) together, we find a dependence of the thickness of the frozen layer on the amount of heat removed during preliminary freezing. The data obtained are presented to construct graphically the dependence of the thickness of the frozen layer on the amount of heat (Figure 4).

As a result of processing of the experimental data, a semi-empirical dependence is obtained, which is presented in the form of Formula (28):

(28) $$Q_{pre.fr}=-6.9e-0.07\cdot \delta^{3}+1.3e-0.05\cdot \delta^{2}-1.6e-0.05\delta +13.3,$$

where

$Q_{pre.fr}—\;$the amount of heat in the pre-frozen layer of the product, J;

δ—the thickness of the frozen layer, mm.

The dependence of the thickness of the frozen layer on the amount of heat removed during preliminary freezing has a cubic character.

Investigating the dependence of the thickness of the frozen layer on the amount of heat removed during preliminary freezing to an extremum, it was found that the minimum thickness of the pre-frozen layer, which ensures the absence of adhesion when freezing chopped squash cubes into single block, lies in the range 0.4–0.9 mm.

Simultaneously, with the change in the phase state, namely, the freezing of the surface of squash cubes, the adhesive properties of their surface decrease, which also reduces the possibility of the sticking of squash cubes into one block.

It is rational to use a cascade device for freezing, as offered by us as one of the elements of freezing lines for juicy fruit and vegetable products and sliced products.

In a cascade freezer, fruits or vegetables are continuously mixed. At the same time, low-temperature air cools them to cryoscopic temperature, and then freezes the surface layer. Due to this, the pieces cease to stick together. The frozen surface layer also protects the fruit from damage, forming a protective coating.

Thus, the prepared product can be frozen in conventional refrigeration equipment.

**Figure 5:**
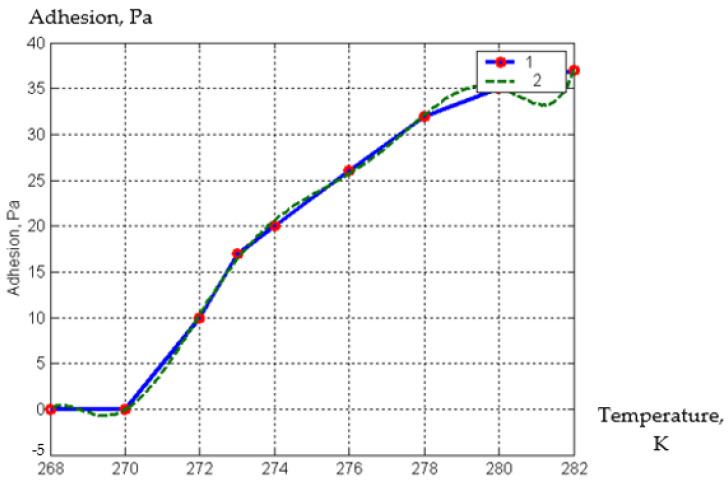
The dependence of the adhesion (Pa) of pieces of squash to each other on the temperature (K) on their surface; 1—experimental curve, 2—approximating curve.

As a result of the determination of the temperature at which the adhesion of pieces of squash in one block ceases, the dependence was obtained (Figure 5).

As a result of processing the experimental data, a semi-empirical dependence was obtained, which is presented in the form of Formula (29):

(29) $$Q_{pre.fr}=0.00011\cdot T^{7}-0.21\cdot T^{6}+1.7e+0.02\cdot T^{5}-7.8e+0.04\cdot T^{4}+2.1e-0.03\cdot T^{3}+3.5e+0.09\cdot T^{2}+3.2e+0.11\cdot T-1.3e+0.13\;\;,$$

where

T—current temperature of the frozen product, K.

It can be seen from the graph that adhesion practically ceases at a temperature of minus 3–5 °C. This fact provides additional information on the temperature sufficient for preliminary freezing of the surface layer of squash cubes.

We also investigated the temperature change in the freezing of squash over time. This data are necessary to determine the residence time of the product pieces in a cascade freezer. The length of time the product slices are in the cascade freezer is determined by the temperature change schedule.

This is equal to the time the sample needs to reach a temperature 2–3 degrees below cryoscopic temperature.

In graph 7, the cryoscopic temperature is clearly visible as a direct horizontal isotherm.

For this, thermocouples were installed on the surface and at various depths from it, up to the center of the sample, and the product was placed in a cooling medium.

In this experiment, thermocouples were installed on the surface and at different depths from it, to the center of the sample, and the product was placed in a cooling medium. What interests us in this case is precisely the temperature dependence of only the top layer when freezing pieces of squash against time at the temperature of the freezer, which is presented in the graph.

**Figure 6:**
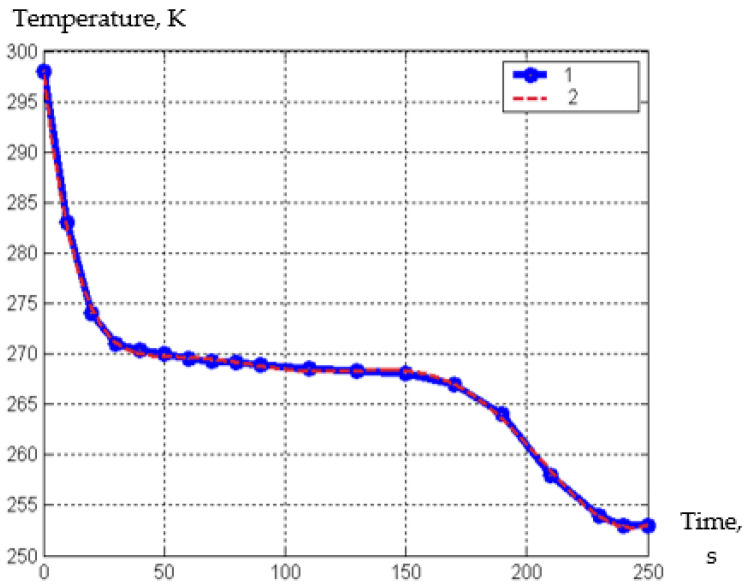
The temperature (K) dependence of the upper layer when freezing pieces of squash from time (s), to the time at a freezer temperature of −25 °C; 1—experimental curve, 2—approximating curve.

The results of the studies are presented in Figure 6.

As a result of processing the experimental data for freezing pieces of squash in air, a semi-empirical dependence is obtained, which is presented in the form of Formula (30):

(30) $$T=-8.5e-0.14\cdot \tau^{7}+8.6e-0.11\cdot \tau^{6}-3.4e-0,08\cdot \tau^{5}+7.1e-0.06\cdot \tau^{4}-0.00081\cdot \tau^{3}+0.052\cdot \tau^{2}-1.8\cdot \tau +3.002$$

where, *τ*—time, s.

It can be seen from the graph 6 that the period in our cascade device for freezing juicy fruits, berries, or sliced vegetables lasts 5–10 min, until the formation of an ice crust on their surface. Further freezing can be carried out in conventional refrigeration equipment.

**Figure 7:**
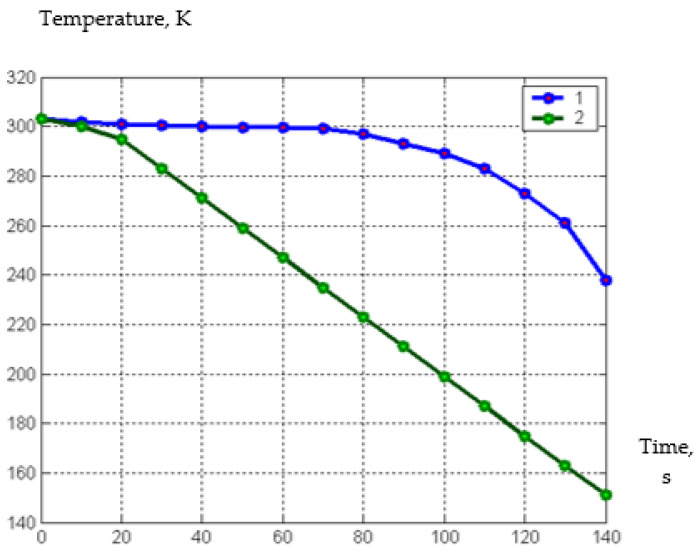
The temperature (K) dependence of freezing of squash pieces on time (s) with a freezing agent in nitrogen vapor and in liquid nitrogen; 1—freezing pieces of squash in nitrogen vapor, 2—freezing pieces of squash in liquid nitrogen.

The time in the cascade freezer for our juicy fruits, berries, or chopped vegetables can be significantly reduced if other refrigerants are used instead of air. Therefore, we investigated the freezing of juicy berries, fruits, and chopped vegetables in nitrogen vapors and in liquid nitrogen. The results of the study are presented in Figure 7.

As a result of processing the experimental data for freezing pieces of squash in nitrogen vapor, a semi-empirical dependence was obtained, which is presented in the form of Formula (31):

(31) $$T=-5e-0.06\cdot \tau^{4}+6.9e-0.05\cdot \tau^{3}-0.0029\cdot \tau^{2}-0.025\cdot \tau +3.002$$

As a result of processing the experimental data for freezing pieces of squash in liquid nitrogen, a semi-empirical dependence was obtained, which is presented in the form of Formula (32):

(32) $$T=-7.9e-0.09\cdot \tau^{5}-3.4e-0.06\cdot \tau^{4}+0.00054e-0.04\cdot \tau^{3}-0.04\cdot \tau^{2}-0.15\cdot \tau +3.002$$

An analysis of the obtained formulas and graphs shows that the formation time of the ice layer on the surface of the pieces in nitrogen vapors, which can be determined by passing zero degrees and cryoscopic temperature, is two minutes; and even less in liquid nitrogen, only 25–30 s.

## 4. Conclusions

Application of the cascade freezer of the proposed design, by installing a mesh hollow rotating drum, and the implementation of a mesh conveyor in the form of a vibrating conveyor with eccentric mechanism and springs, allows simplifying the design, reducing energy consumption, improving the stability of the fluidized bed of the product being frozen, and increasing the intensity of heat transfer in the fluidized bed and the performance of the freezing process as a whole.

We presented a model or algorithm for calculating energy costs for fluidization and mechanical throwing. A model for freezing the product cube was presented.

The energy costs for creating a fluidized bed compared to mechanical shaking during freezing raspberries are 1.5–3.5-times higher.

This methodology for determining the energy cost criteria and the main design parameters of a cascade freezer can be used in future designs.

We offer a cascade freezer, as an option for improving the refrigerating process equipment for freezing small-sized products in a dense fluidized bed, and which is of practical importance and can be used in production.

## Figures and Tables

**Table 1:** Thermophysical characteristics of squash.

Parameter	Designation	Meaning
Temperature, K:		
initial	*t_i.e.,_*	298
cryoscopic	*t_cr_*	272
freezing medium	*t_e._*	233
Specific heat capacity, kJ/(m^3^·K):		
at positive temperatures	*c_p_*	3.8
at low temperatures	*c_fr_*	2.3
Density, kg/m^3^:		
at positive temperatures	*ρ*	940
at low temperatures	*ρ_fr_*	700
Specific heat of fusion, kJ/kg	*λ_mlt_*	333
Coefficient of thermal conductivity, W/(m·K):		
at positive temperatures	*λ_p_*	0.23
at cryoscopic temperature	*λ_cr_*	0.2
at low temperatures	*λ_fr_*	1.5

**Table 2:** Distribution of thermophysical parameters versus temperature.

Parameter	Temperature, K					
	273	268	263	258	253	248
The coefficient of thermal conductivity, W/(m·K)	0.21	1.23	1.34	1.43	1.53	1.56
Specific heat capacity, kJ/(m^3^·K)	3.8	2.3	2.27	2.23	2.22	2.21
Density, kg/m^3^	940	870	836	821	770	700

## Data Availability

Data sharing not applicable.
